# Patient-specific scaphoid prosthesis: surgical technique

**DOI:** 10.1007/s00402-024-05609-7

**Published:** 2024-12-16

**Authors:** Philipp Honigmann, Joris Oonk, Johannes Dobbe, Simon Strackee, Geert Streekstra, Mathias Haefeli

**Affiliations:** 1 Hand and Peripheral Nerve Surgery, Department of Orthopaedic and Trauma Surgery, Kantonsspital Baselland, Bruderholz, Laufen, Liestal, Switzerland; 2 Biomedical Engineering and Physics, Amsterdam UMC Location University of Amsterdam, Meibergdreef 9, Amsterdam, The Netherlands; 3 Amsterdam Movement Sciences, Musculoskeletal Health - Restoration and Development, Amsterdam, The Netherlands; 4 Department of Plastic, Reconstructive and Hand Surgery, Academic Medical Center, University of Amsterdam, Amsterdam Movement Sciences, Amsterdam, The Netherlands; 5 Hand Surgery, Kantonsspital Graubünden, Chur, Switzerland

**Keywords:** Scaphoidreplacement, Scaphoidprosthesis, Patient-specific, 3D-printing, Salvage, Wrist

## Abstract

The scaphoid bone is essential for wrist stability, force transmission, and movement, being crucial for maintaining carpal kinematics. In cases where the scaphoid is non-reconstructable, a complete replacement can serve as a treatment option to preserve carpal alignment and motion. This approach has evolved since its first descriptions in 1945, benefiting significantly from advancements in patient-specific implant design, additive manufacturing/3D printing, and material use in recent years. We present a technique for scaphoid replacement using a patient-specific prosthesis and reconstruction of intrinsic and extrinsic ligaments to achieve optimal stability and mobility.

## Introduction

Complete replacement of the Scaphoid can be a treatment option in cases of a non-reconstructable Scaphoid, to maintain carpal motion and restore carpal kinematics [1]. First descriptions reach back to 1945 [2].

Due to the anatomical variability of the Scaphoid, an individualized patient-specific replacement is favourable [3]. In recent years, the development of patient-specific implants (PSI) for replacing the Scaphoid has been advanced in terms of design, manufacturing and material use [4–7].

The concept of patient-specific implants involves the utilization of advanced imaging techniques, such as computed tomography (CT) or magnetic resonance imaging (MRI), to generate an accurate and precise 3D replacement model of the patient's affected bone mainly based on the mirrored healthy contralateral side [7–10]. This model serves as the basis for designing and fabricating a custom-made implant that matches the patient's unique carpal anatomy. A functional suspension of the prosthesis is required to restore function and normal carpal kinematics. Care must be taken to avoid adaptation of the carpal bone geometries. So far, the prostheses have been used as spacers only and were initially only placed in the former space of the scaphoid. Spingardi and Rossello used the last version of the Swanson Titanium implant which has been fixed to the lunate with an anchor and with a sort of spine into the proximal trapezium [1]. The latter blocks the scapho-trapezio-trapezoidal (STT)-joint which we believe is a key joint to allow for motion of the scaphoid especially in radial and ulnar deviation. In a first cadaveric study in 2018 we used a technique to suspend the prosthesis based on the reconstruction of the scapho-lunate ligament described by Henry [4, 11].

We modified this suspension technique and provide a detailed technical description of the preoperative planning process and the creation of a patient-specific implant design based on CT-scans and Digital Imaging and Communications in Medicine (DICOM) data. The surgical procedure itself is then described in detail. Postoperative care, rehabilitation protocols, and potential complications will also be addressed.

## Anatomy

The scaphoid bone is a vital component of the wrist, playing a crucial role in stability, force transmission, and joint movement.

The Scaphoid is located in the proximal carpal row comprising a body, proximal pole, distal pole, a tubercle, and a waist. It articulates with the scaphoid fossa of the distal radius, the lunate, capitate, trapezium and trapezoid. Biomechanically, the scaphoid contributes to wrist stability, load transmission, and wrist function. It enables flexion, extension and radial/ulnar deviation. The interplay between the scaphoid, neighbouring carpal bones, and ligaments facilitates coordinated wrist movements. Strong ligamentous connections especially of the dorsal aspect of the scapho-lunate ligament as well as the scapho-capitate and the dorsal intercarpal ligament provide stable intrinsic and extrinsic connections [12, 13]. This strong connection between the first and second carpal row is essential to maintain carpal alignment [14]. Uncoupling of the carpal rows and carpal collapse are the sequelae of injuries of the scapho-lunate ligament and especially of scaphoid non-unions [15]. The persistence of carpal collapse leads to degenerative changes known as scapho-lunate advanced collapse (SLAC) and scaphoid non-union advanced collapse (SNAC) [16, 17].

**Fig. 1:**
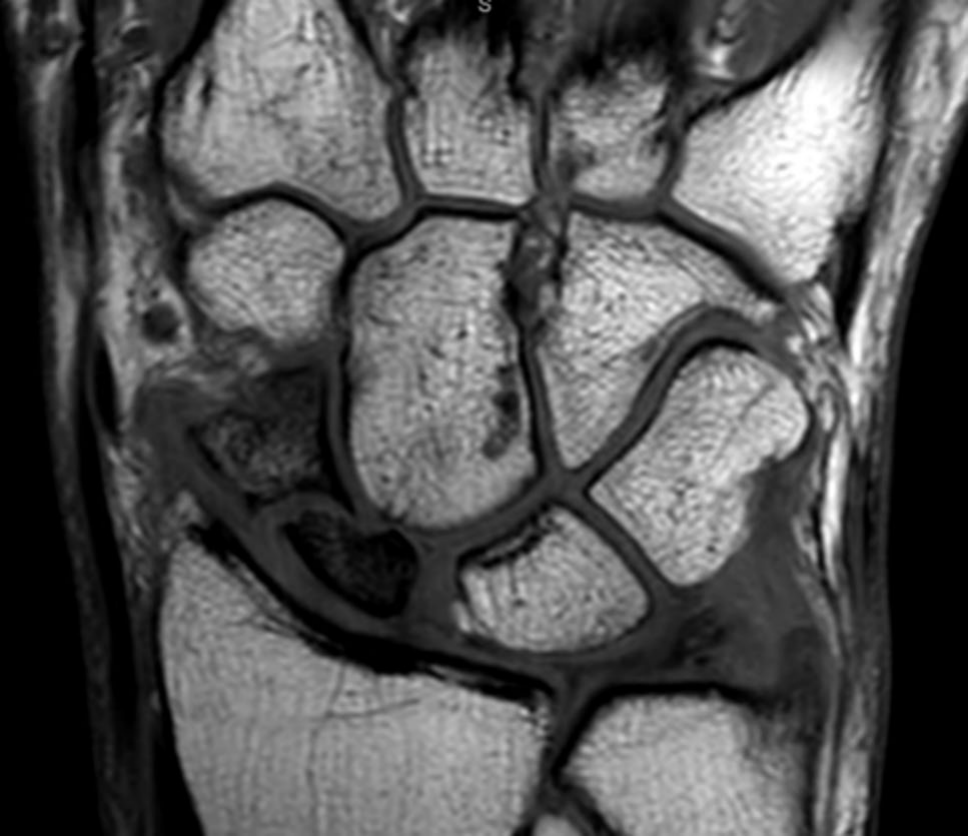
Avascular necrosis of the Scaphoid pole due to non-union

**Fig. 2:**
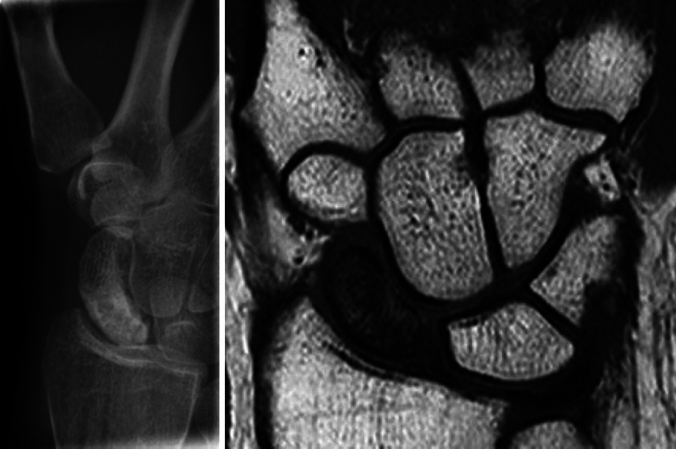
Preiser's disease (Stage III)

Vascular supply to the scaphoid is primarily through the dorsal and palmar branches of the radial artery which supplies an average of 83% of the scaphoid’s blood volume [18]. Morsy and colleagues identified two morphological bone types based on the vascular network: type I or full scaphoids and type II or slender scaphoids [18]. Type I possessed a more robust internal vascular network than type II scaphoids. Type II seems to be more vulnerable to the development of avascular necrosis (AVN) like in non-union (Fig. 1) or Preiser’s disease (Fig. 2) [19].

The size and volumes of the scaphoid varies significantly between males and females. Heinzelmann et al. found a variation of 31.3 ± 2.1 mm vs. 27.3 ± 1.7 mm in length and width at the proximal pole of 4.5 mm ± 1.4 vs. 3.7 mm ± 0.5 and at the waist 13.6 mm ± 2.6 vs. 11.1 mm ± 1.2 respectively [20]. Pichler et al. examined 30 scaphoids in 30 healthy wrists and mentioned a mean volume of 3389.5 mm^3^ and found a significantly higher volume in men (4057.87 mm^3^) than in women (2846.57 mm^3^) with a high variation in volumes in their cohort [21]. This clearly marks the need for an individualized approach using a patient-tailored prosthesis when aiming for a replacement of the Scaphoid.

## Indications/contraindications for scaphoid replacement

**Fig. 3:**
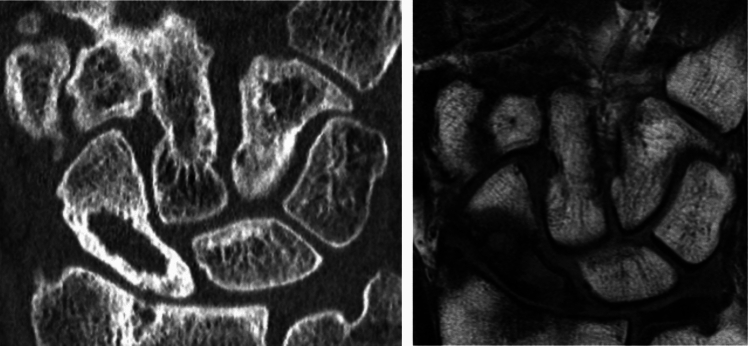
destroyed scaphoid due to infection after percutaneous screw fixation of a B2-type fracture

**Fig. 4:**
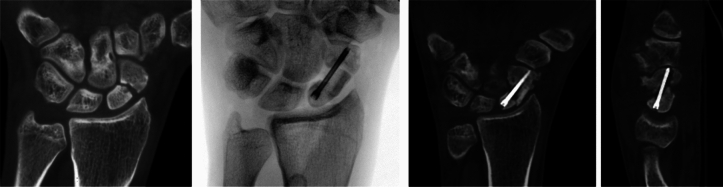
course of a failed reconstruction with screw loosening and persistent non-union

**Fig. 5:**
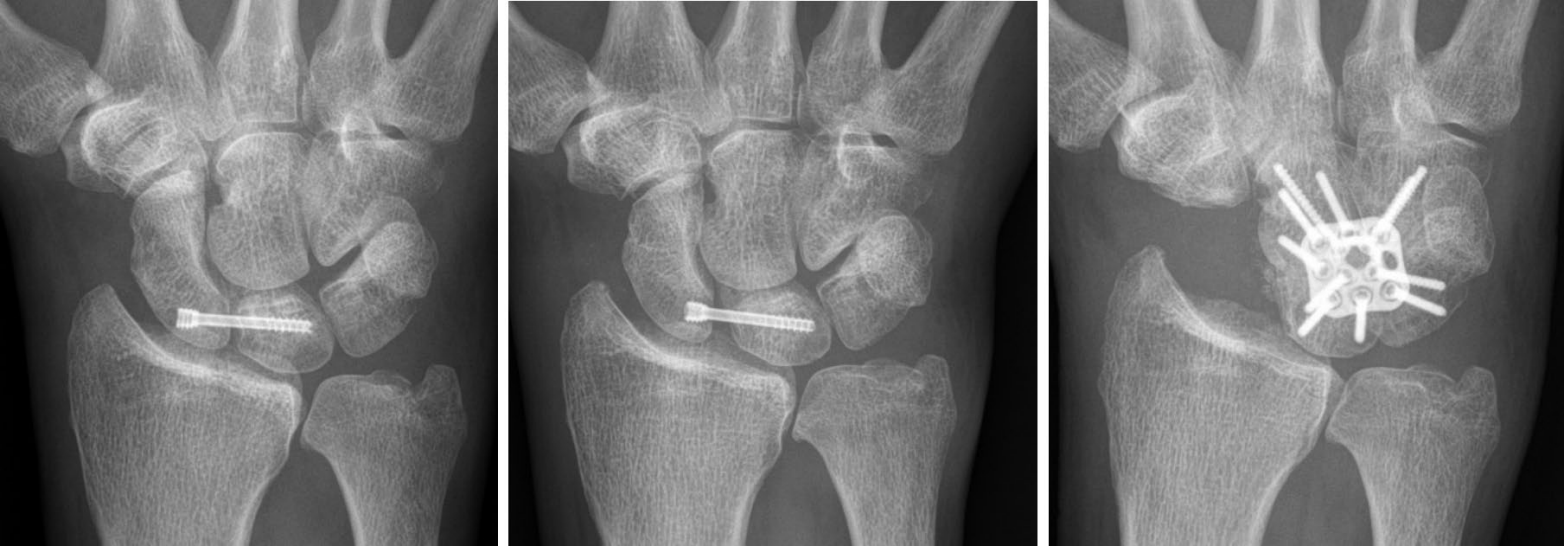
Course of a screw loosening of a RASL-procedure. Left to right: initial screw fixation, screw migration, four-corner fusion of the capitate, lunate, triquetrum and hamate

An appropriate indication is the destroyed Scaphoid either due to failed osteosynthesis (Fig. 3) or failed reconstruction (Fig. 4), sequelae of failed Reduction and Association of Scaphoid and Lunate (RASL) procedure (Fig. 5), being unsuitable for reconstruction techniques or requiring rescue interventions such as proximal row carpectomy (PRC) or partial wrist (i.e. 4-corner) fusion [1, 22–25].

Spingardi and Rossello state that, for a good outcome of a scaphoid replacement with a prosthesis, good wrist stability and absence of degenerative change in the radiocarpal or midcarpal joint are required [1]. Correspondingly, the authors listed the following contraindications: radial scaphoid facet degeneration; a previously performed radial styloidectomy; any sign of carpal collapse and deformity of the distal radius consequent to displaced fracture; a diminution of the carpal height; any increase in the radiolunate angle; or degenerative arthritis of other carpal bones, in particular at the midcarpal joint. The authors expanded and structured this list incorporating additional indications and contraindications, which can be found in Tables 1 and 2.

**Table 1:** Indications

Posttraumatic/degenerative	Postinterventional/iatrogenic	Disease associated
Complex scaphoid non-union	Failed osteosynthesis	Avascular necrosis of scaphoid (Preiser's disease)
Non-reconstructable destroyed scaphoid	Failed reconstruction	Congenital scaphoid pathologies associated with symptomatic carpal instability (i.e. bipartite scaphoid)
Postinfectious destruction	Failed reduction and association of SL with non-reconstructable scaphoid

**Table 2:** Relative and absolute contraindications for scaphoid replacement

Relative contraindication	Absolute contraindication
SNAC/SLAC II	SNAC/SLAC III/IV
Bisphosphonate therapy (increased risk of AVN of the entire proximal row) [21]	Concomitant AVN of the scaphoid and lunate in thalassemia minor patients [22]
	Diseases affecting the lunate (i.e. Kienböck’s disease) [23, 24]
	Skeletally immature patients

## Method

### Biomechanical considerations

**Fig. 6:**
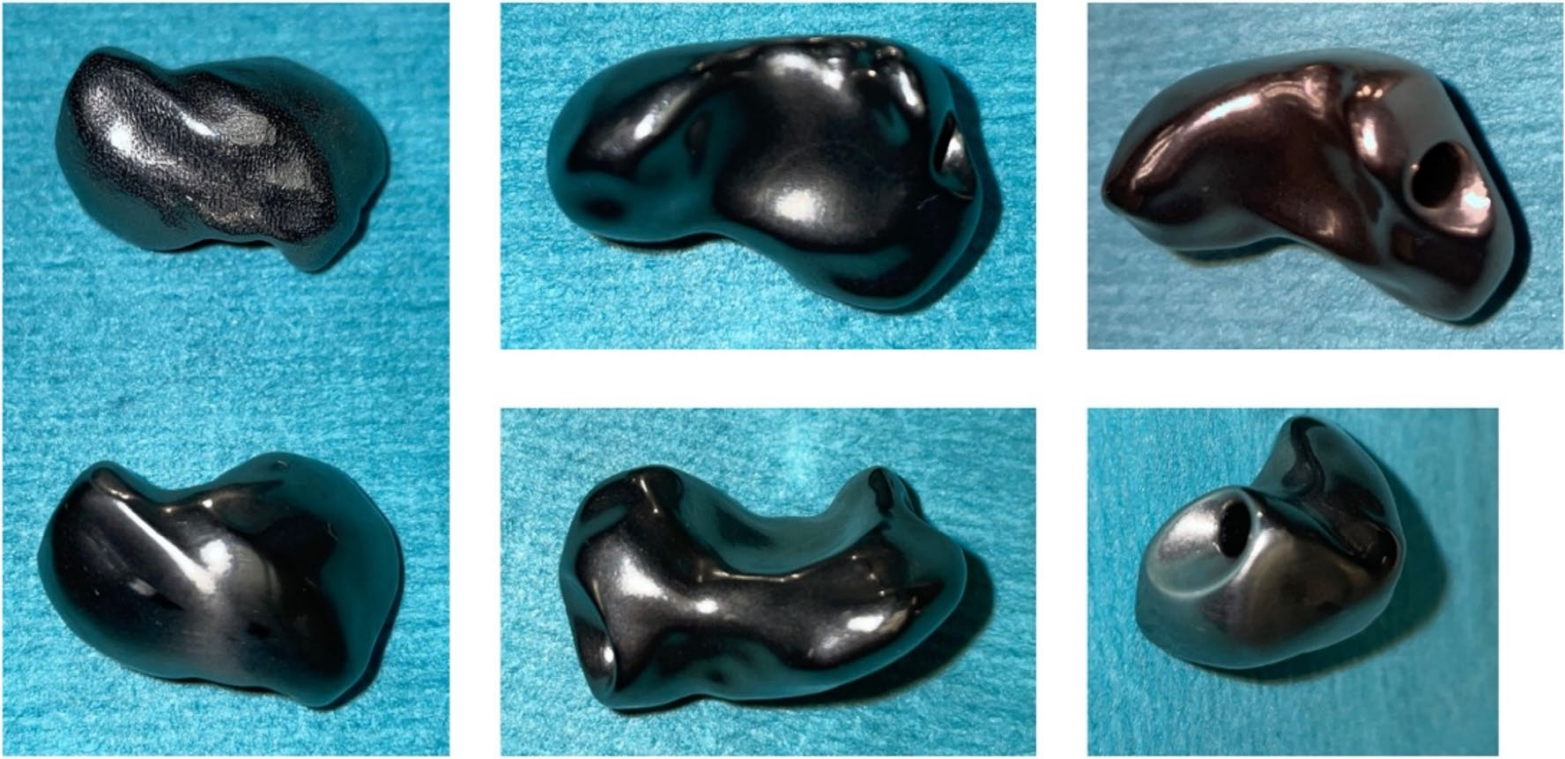
the patient-specific scaphoid prosthesis made of 3D-printed Titanium

The primary stability of the prosthesis is achieved through its customized design, ensuring an optimal fit. To facilitate motion at the STT-joint, the distal palmar entrance of the prosthesis channel is adapted to have a funnel-shaped design providing more play in the ligament-prosthesis interface to allow for more motion of the prosthesis (Fig. 6).

To enable normal carpal motion, the intrinsic and extrinsic ligaments are reconstructed to serve as primary and secondary ligamentous stabilizers, effectively suspending the prosthesis. Our choice of suspension technique follows the scapholunate ligament reconstruction method described by Sandow et al., known as anatomical front and back repair (ANAFAB) [26]. This technique allows for an anatomical reconstruction of both the anterior and posterior aspects of the scapholunate complex.

### Design and production of the prosthesis

The design is based on a mirrored 3D computed tomography (CT) scan of the contralateral wrist using the mirrored contralateral healthy scaphoid as the design model and, in cases where both sides are affected, utilizing statistical shape models (CMX Medartis, Basel, Switzerland) [10]. Several factors during the planning process need to be considered, including side differences, the thickness of the cartilage surface layer and potential errors that may occur during segmentation, a process where medical images are divided into different regions or structures to better analyze and visualize specific tissues, such as bone, cartilage, or soft tissues, for the purpose of treatment planning or diagnosis [27]. To facilitate movement in the STT-joint, the entrance of the curved channel is designed in a funnel-like shape. The edges of both the entrance and exit of the channel are rounded to prevent any disruption of the tendon/graft suspension. The prosthesis is manufactured from Titanium powder (Ti-6Al-4V Grade 23, according to ASTM F 3001 / F136 regulations) using selective laser melting (SLM, Sisma MYSINT 100 RM), a 3D printing technique. Alternatively, milling is an option, but it results in a V-shaped design for the channel due to limitations in the drilling process. To ensure optimal function, the prosthesis must be carefully designed, considering variations in size and material during the design and production process. Therefore, entrusting the design and manufacturing process to certified medical engineers and medical technology companies is crucial (CMX Medartis, Basel, Switzerland and Swiss m4m Center, Bettlach, Switzerland). If the prosthesis is too large, it can result in stiffness and deterioration. Insufficient surface postprocessing may lead to the erosion of the cartilage layer of surrounding bones.

## Surgical technique

### Approach

**Fig. 7:**
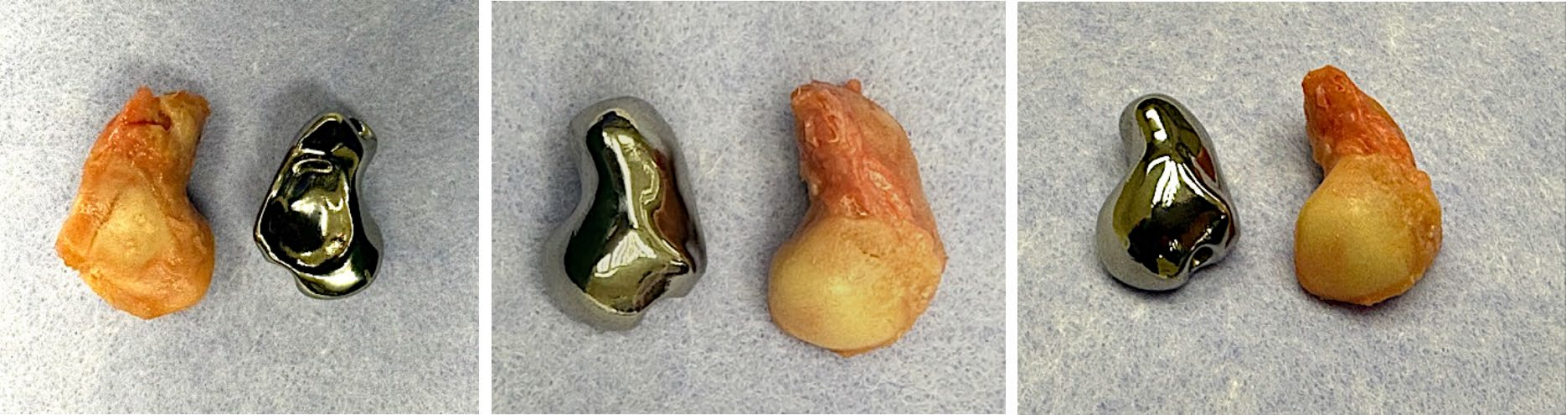
comparison between the native scaphoid and the PSI-prosthesis

To assess degenerative changes in the radiocarpal and midcarpal joints, we perform an arthroscopy as the first step before designing the implant. The second surgical intervention is performed using a dorsal approach to the radius and carpus through to the third extensor compartment. The second and fourth extensor compartments are elevated followed by a neurectomy of the posterior interosseous nerve (PIN). A distally based capsular flap is raised, and the dorsal intercarpal ligament is detached from its insertion points at the scaphoid and lunate. This procedure involves mobilizing the scaphoid and separating it from the scapholunate and the strong palmar radioscaphocapital ligaments. The scaphoid can then be successfully extracted (Fig. 7).

### Suspension

**Fig. 8:**
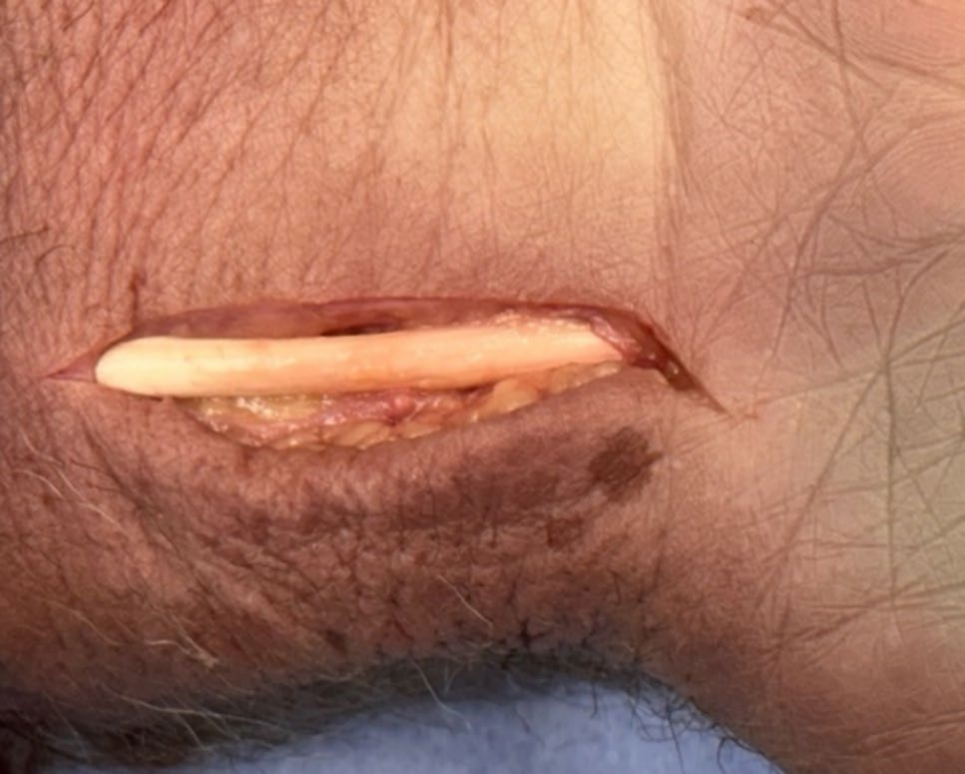
volar modified Henry approach

Following a modified volar Henry approach (Fig. 8), the sheath of the flexor carpi radialis tendon (FCR) is opened and the radial third (2—2.5 mm) is separated from the tendon [28].

**Fig. 9:**
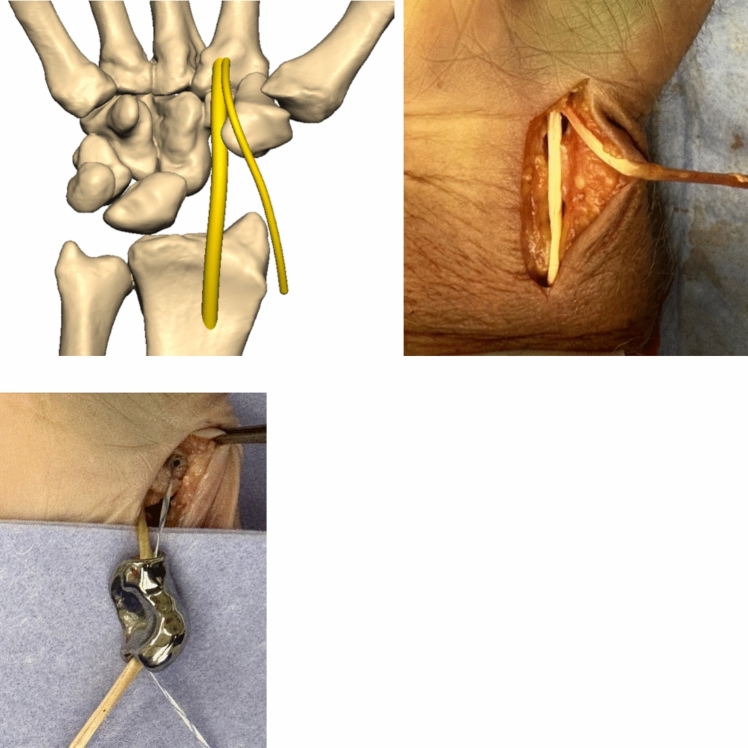
mounted prosthesis with elevated FCR-Tendon strip and FibreTape® being attached to the Trapezium

The graft is then elevated proximally at the level of the musculotendinous junction leaving the distal insertion intact. Like in the ANAFAB-technique a FibreTape® (Arthrex, Naples, Florida, USA) is secured to the radial trapezium facet using a 2.4 PushLock®-Anchor (Arthrex, Naples, Florida, USA) (Fig. 9).

**Fig. 10:**
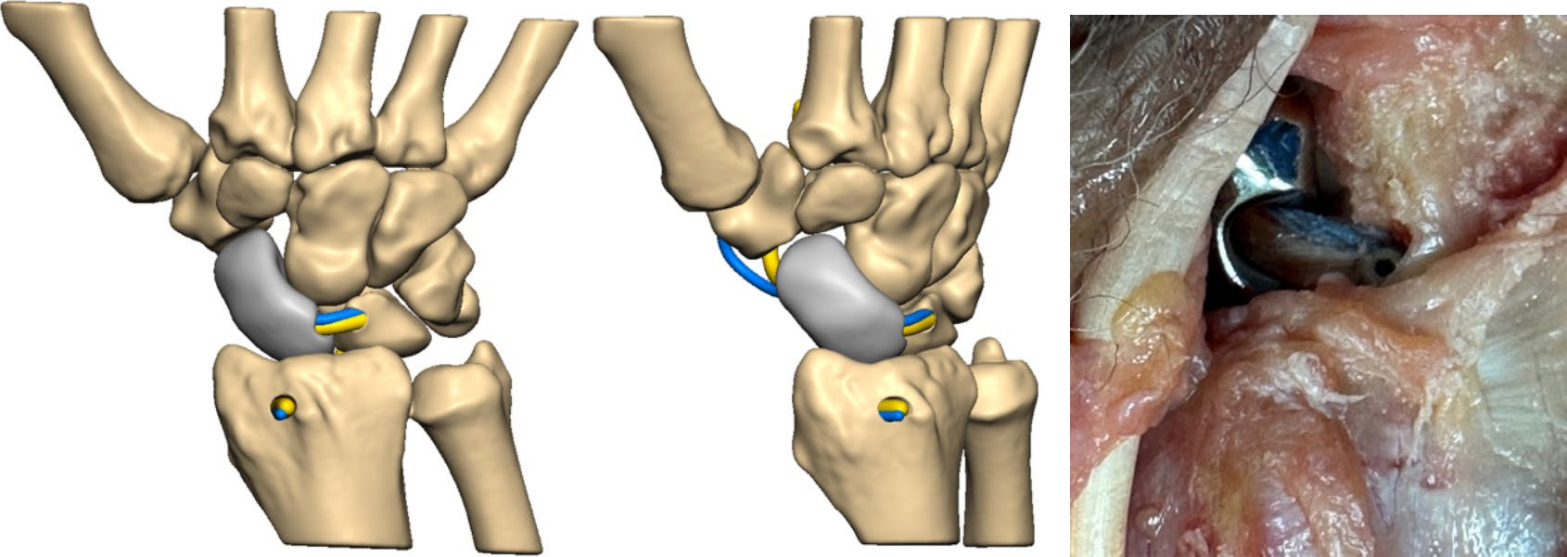
final situation with the view of the reconstructed scapholunate ligament (FCR-strip = yellow and FibeTape® = blue)

**Fig. 11:**
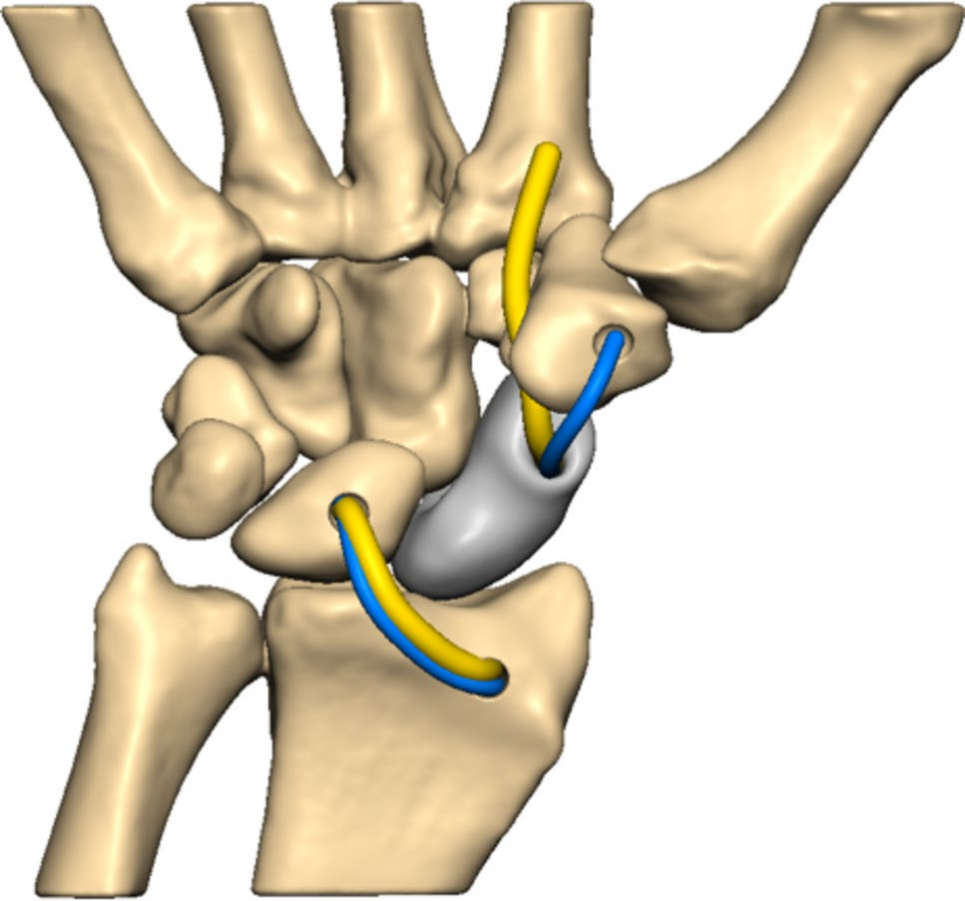
reconstruction of the long radiolunate ligament (LRL) with channel in the distal radius to attach the LRL

To establish proper fixation, a 3.0 mm oblique channel is drilled from the dorsoradial corner to the volar side of the lunate, aligning with the insertion of the long radiolunate ligament (LRL), using a K-wire and cannulated drill. Additionally, a 2.7 mm channel is created from the palmar to dorsal aspect of the radius at the origin of the LRL (Fig. 10). Both the FCR-tendon strip and the FibreTape® are passed through the prosthesis from the palmar to the dorsal side, and then back through the lunate channel. A 3 × 8 mm Biotenodesis-Screw® (Arthrex, Naples, Florida, USA) is inserted into the channel from dorsally to uncouple the dorsal SL-ligament reconstruction from the LRL (Fig. 11). The construct is carefully tightened and again both the FCR-tendon strip and the FibreTape® passed from the palmar to the dorsal aspect through the channel into the radius. Finally, a 3 × 8 mm Biotenodesis-Screw® is inserted into the channel to secure the construct.

### Postoperative treatment

Hand therapy begins on the first day after surgery where a thermoplastic splint is applied and active-assisted mobilization is initiated two weeks postoperatively within a range of 30° flexion and extension, and 30° pro-/supination. However, radial and ulnar deviation of the wrist should be avoided for the first six weeks. Following clinical and radiological assessments, six weeks postoperatively, the thermoplastic splint is replaced with a more flexible wrist brace and unrestricted motion out of the brace is allowed. Gradual increase in weight bearing is introduced between the 7th and 12th weeks to achieve full motion and weight bearing.

## Expected outcome

The implant allows for a full range of motion and weight bearing. The individual results depend on dexterity, constitution and preoperative pathology and amount of procedures performed before. Patients with the first successful implantations gained full weight bearing after 3 months and similar range of motion in flexion/extension, pro-/supination and radial-/ulnarduction.

**Fig. 12:**
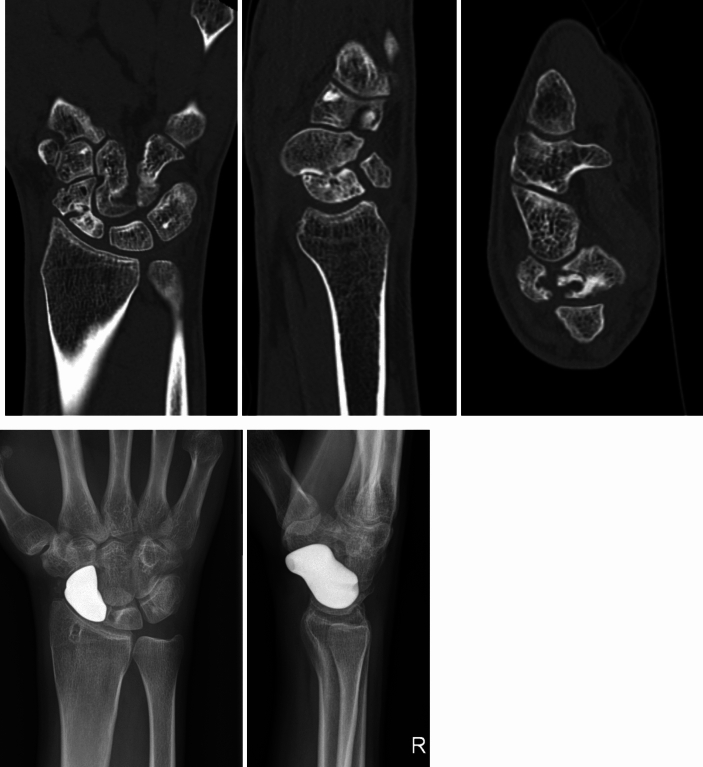
CT-scans of a complex scaphoid non-union 6 years after initial trauma and implanted scaphoid prosthesis

Figure 12 shows the X-rays of a patient who sustained a Scaphoid fracture with 24 years of age. He presented with a complex non-union 6 years later with the age of 30. We decided to implant a patient-specific prosthesis. The patient regained a good range of motion and grip strength of the healthy contralateral side. The patient related outcomes are shown in Table 3.

**Table 3:** patient data (active range of motion and grip strength before and after scaphoid replacement)

	Left side (healthy)	Right side (pre-operative)	Right side (6 months post-operative)
Flexion/extension [°]	80/0/70	55/0/50	50/0/50
Pro-/supination [°]	90/0/80	90/0/70	80/0/90
Radial-/ulnarabduction [°]	35/0/45	25/0/35	20/0/40
Grip-strength [kg]	44	30	44

## Complications

In addition to common issues such as infection, haematoma, and wound healing difficulties, there are also specific complications related to prostheses and their suspension.

Excessive tightness in the suspension can restrict the freedom of movement. To ensure adequate mobility of the prosthesis, it is important to leave enough room for the tendon / synthetic tape combination in the STT-joint. To prevent lunate fractures or necrosis, it is crucial to ensure that the suspension is not too tight, that the channel in the lunate is precisely aligned and does not exceed a diameter of 3.0 mm, as in the ANAFAB approach.

Inadequate suspension, which can occur due to anchor loosening or wear and tear of the suspension tendon/synthetic tape construct, may lead to the dislocation of the prosthesis.

Regarding the durability, there has never been a report on breakage or desintegration of a titanium prosthesis. It’s a solid body printed with Titanium powder (Ti_6_Al_4_V) using a selective laser melting (SLM) process. The production is ISO 13485 certified which includes material checks and an implemented quality management system.

## Discussion

It is well known that the scaphoid has a inter-individual variation in size and shape which also contributes to an individual motion pattern [12, 13, 29]. Even though Scaphoid replacement has a long history of using various materials such as vitallium, titanium and silicone, a patient-specific replacement has not been considered until 2018 [2, 4, 30, 31]. One of the key advantages of utilizing a patient-specific implant (PSI) is the patient-individual anatomical fit. The custom design of the prosthesis based on the patient's contralateral side remains the gold standard to ensure an optimal match, anatomical alignment and primary stability even though there are small differences in size [27]. When both sides are affected, a statistical shape model could be used. Another aim of the proposed technique is to allow for the restoration of normal wrist biomechanics as a prerequisite for better joint function and increased range of motion. The latter needs to be confirmed in clinical studies.

We believe that reconstructing the intrinsic and extrinsic ligaments plays a crucial role in achieving a natural carpal motion pattern and restoring function. This is particularly important in cases where there is early carpal collapse and dorsal intercalated segment instability (DISI) of the lunate bone. By restoring the long radiolunate ligament using an oblique channel through the lunate, it becomes possible to correct the DISI deformity and promote improved alignment. That is the reason why we chose the ANAFAB-technique for the reconstruction of the scapholunate ligaments [26].

Disadvantages of the patient-specific design are the time-intensive individual design and production process and the manual post-production steps such as polishing. The design and manufacturing of PSI require advanced imaging techniques and adherence to a certified manufacturing process according to CE and ISO standards, which can be financially demanding. The manufacturing process costs about 1′500–2′000 €. The surface needs to be as smooth as possible to avoid any destruction of the cartilage of adjacent joint surfaces. In-vitro wear-out tests and long-term in-vivo studies are necessary to investigate any interference of the prosthesis with the cartilage and to evaluate other above-mentioned materials. Using other implantable biocompatible materials such as Poly-Ether-Ether-Ketone (PEEK) or ultra-high-molecular weight polyethylene (UHMWPE) may be used for implant production and may also simplify the post-production processes [5, 6].

In conclusion, the replacement of the scaphoid using a patient-specific prosthesis with a reconstruction of the extrinsic and intrinsic ligaments is an innovative surgical technique that offers a potential benefit in the treatment of non-reconstructable scaphoid bones. This technique does not burn any bridges for possibly necessary future salvage procedures like proximal row carpectomy, wrist arthroplasty and partial (four corner) or complete wrist fusion.

## Data Availability

No datasets were generated or analysed during the current study.
